# Effects of the application of microbiologically activated bio-based fertilizers derived from manures on tomato plants and their rhizospheric communities

**DOI:** 10.1038/s41598-023-50166-5

**Published:** 2023-12-18

**Authors:** Elisa Clagnan, Mirko Cucina, Patrizia De Nisi, Marta Dell’Orto, Giuliana D’Imporzano, Roberto Kron-Morelli, Laia Llenas-Argelaguet, Fabrizio Adani

**Affiliations:** 1 Gruppo Ricicla Labs., Dipartimento di Scienze Agrarie e Ambientali—Produzione, Territorio, Agroenergia (DiSAA), Università Degli Studi Di Milano, Via Celoria 2, 20133 Milano, Italy; 2 Department for Sustainability, Biotechnologies and Agroindustry Division, ENEA, Italian National Agency for New Technologies, Energy and Sustainable Economic Development, Casaccia Research Center, Via Anguillarese 301, 00123 Rome, Italy; 3 National Research Council of Italy, Institute for Agriculture and Forestry Systems in the Mediterranean (ISAFOM-CNR), Via Della Madonna Alta 128, 06128 Perugia, Italy; 4 Agrifutur Srl, Via Campagnole 8, 25020 Alfianello, BS Italy; 5 BETA Tech Center, TECNIO Network, University of Vic-Central University of Catalonia, Ctra de Roda 70, 08500 Vic, Spain

**Keywords:** Microbiology, Environmental sciences

## Abstract

Bio-based fertilizers (BBFs) recovered from animal manure are promising products to optimise resources recovery and generate high agricultural yields. However, their fertilization value may be limited and it is necessary to enrich BBFs with microbial consortia to enhance their fertilization value. Three specific microbial consortia were developed according to the characteristics of three different BBFs produced from manure (bio-dried solid fraction, solid fraction of digestate and biochar) to enhance plant growth and product quality. A greenhouse pot experiment was carried out with tomato plants grown with microbiologically activated BBFs applied either as N-organic fertilizers or as an organic amendment. A next generation sequencing analysis was used to characterise the development of each rhizospheric community. All the activated BBFs gave enhanced tomato yields (fresh and dry weight) compared with the non-activated treatments and similar to, or higher than, chemical fertilization. Concerning the tomato fruits’ organoleptic quality, lycopene and carotenoids concentrations were improved by biological activation. Metagenomic analysis points at *Trichoderma* as the main driver of the positive effects, with the effects of added bacteria being negligible or limited at the early stages after fertilization. In the context of the circular economy, the activated BBFs could be used to replace synthetic fertilisers, reducing costs and environmental burdens and increasing production.

## Introduction

Synthetic fertilizers are currently the basis of modern agricultural systems where their use is required to increase productivity and sustain global food demand. These synthetic fertilizers are generally derived from mined resources (phosphorus and potassium) or industrial processes (nitrogen). The production of synthetic fertilizers relies therefore on fossil energies and non-renewable raw materials, leading to the high increase of prices in recent years^1^. As an example, the price of urea increased by about four times in one year (spring 2021 – spring 2022) due to the COVID-19 pandemic and ongoing conflicts, posing serious threats to the economic sustainability of agricultural production^2^. Organic fertilizers have therefore emerged as a cost-effective solution, particularly in the context of the circular economy.

Animal manure can be recovered and transformed into bio-based fertilisers (BBFs) through several processes (e.g. stripping, scrubbing, anaerobic digestion, liquid–solid separation, biodrying and composting)^3^. For instance, Luo et al.^4^ reported that BBFs recovered from pig manure have a great potential as an alternative to synthetic mineral N fertilisers, with the added benefit of delivering more easily available N for crops during dry seasons, especially as global warming is expected to cause more frequent droughts all over the world. Recently, the REcovered Nitrogen from manURE (RENURE) fertilizers that represent a subcategory of BBFs have been proposed^5^ and a full-scale example of RENURE production was recently described. This wasa preassembled containerized treatment plant (TRL 9) with a “plug and play” approach that treats manure continuously and automatically, using a total raw input of 120 m^3^day^−1^, through a series of separation and concentration steps^6^. To be categorized as RENURE, recovered N fertilizers must have a mineral N content above 90% of the total N and therefore be suitable as a potential substitute for synthetic chemical fertilizers. This compliance with RENURE standards in the near future will most likely allow RENURE fertilizers to be used in areas sensitive to N pollution in the context of the Nitrates Directive (91/676/EEC), beyond the N limits of 170 kg ha^−1^. Preliminary positive results were obtained using BBFs and RENURE fertilizers in substitution of N synthetic fertilizers^7^. Reviewed literature shows that BBFs and RENURE fertilizers’ potential is still not being exploited by farmers^1^. This is mainly due to the need to assess the nutrient use efficiency of BBFs and RENURE fertilizers in valid comparisons with synthetic fertilizers, as well as the need to further investigate their environmental impacts^3^. The microbial composition of BBFs may also deserve more attention, as BBFs can contain pathogens, depending on their source and on their production processes^8^. Recovery processes during the production of BBFs might also be detrimental and could potentially eliminate ubiquitarian beneficial microorganisms for soil that were present in the source material, because of high temperatures and/or the presence of toxic compounds at high concentration. Taking these possible drawbacks into consideration and also considering that nutrient release from BBFs may be too slow if compared to synthetic fertilizers, the addition of specified microbial consortia to BBFs is emerging as a tool to enhance their effectiveness for soil fertilization^9,10^. Indeed, BBFs’ enrichment with microbial consortia could enhance their fertilizing potential by favouring nutrient mobilization, organic matter degradation and plant biostimulation^11^. Microbial consortia made with multiple species of bacteria and fungi have been already used in agriculture with various purposes (i.e., biopesticide, biostimulant and biofertilization), but their use is still limited due to a low and inconsistent efficacy^10,12–14^. In this context, plant growth-promoting rhizobacteria (PGPR), will play a pivotal role in the future of sustainable agricultural production, as they promote plant growth by directly improving plants metabolism (e.g. phosphates solubilization, hormones production, N-fixation, increases in uptake of water and minerals), or by influencing the roots microbiome by enhancing the activities of beneficial microorganisms or suppressing the activity of pathogens^15^. Moreover, field application of PGPRs and microbial consortia is still too little studied, as it is hindered by a lack of specialized equipment for the application close to the roots zone and by a possible higher reduction of the live cell content^14^. However, the enrichment of BBFs with microbial consortia appears to be a suitable strategy for taking advantage of the benefits of BBFs such as the reclamation of nutrients and organic matter, reduced environmental impacts, easy application with widespread trucks, and also of microbial consortia application (i.e. mobilization of nutrients, biostimulant effects). . BBFs may be used as the carriers of microbial consortium into the soil, also providing an optimal substrate for microorganisms to survive and proliferate. Few studies can be found in literature about the biological activation of BBFs. Hussain et al.^9^ have used *Bacillus* sp. AZ6 as an inoculant to produce bio-activated organic fertilizers with various formulations, and they found that the biologically activated fertilizer gave the best performance during maize cultivation, showing increased plant height, grain yield, dry matter, crude protein and fibre contents.

The work described here is part of an H2020-EU project which aims to develop, integrate, test, and validate innovative nutrient management strategies to efficiently recover mineral nutrients and other relevant products with agronomic value from animal manure (FERTIMANURE: Innovative nutrient recovery from secondary sources – Production of high-added value fertilizers from animal manure, Grant Agreement N° 862,849; https://www.fertimanure.eu/en/). Within this project, five pilot scale plants have been developed and studied in five different EU countries to recover N, P and K as mineral fertilizers and organic amendments/organic fertilizers, producing various BBFs. The aim of this study was to assess the effects of three BBFs, produced from various animal manures and processes, activated with three different microbial consortia created ad hoc. Three combinations and interactions of BBFs and consortia were tested through a greenhouse pot experiment on tomato plants, with an application technique mimicking that of a field and close-to-the-root application. Then, their fertilizing effect on tomato production was characterized through quantitative and qualitative analyses, while the permanence of the microbial consortia in the soil was tested by NGS.

## Materials and methods

### Bio-based fertilizers

For the purpose of this study, three BBFs were selected from three of the five pilot plants developed within the FERTIMANURE project (Table 1). These were (1) BBF1: bio-dried solid fraction coming from a bio-drying process treating the solid fractions of pig and poultry manure, developed by the pilot plant run by Universitat de Vic—Universitat Central de Catalunya (Spain); (2) BBF2: the solid fraction of digestate produced by anaerobic co-digestion of cattle manure and energy crops developed by the pilot plant run by Wageningen University (The Netherlands)^16^; (3) BBF3: biochar obtained from poultry manure pyrolysis produced by French pilot plant run by the Centre de Recherche Agroenvironnement et Fertilisation (RITTMO).

**Table 1:** BBFs composition (average ± standard deviation (n = 3)) and usage. Composition data are expressed on dry weight basis except for total solids and pH.

	BBF1	BBF2	BBF3
Characterisation			
Total solids (%)	52.8 ± 0.7	26.2 ± 0.4	90.3 ± 1.1
pH (pH unit)	7 ± 0	7.4 ± 0.1	9.1 ± 0.1
Total organic C (%)	17 ± 1	37 ± 2	54 ± 3
Total N (%)	2.8 ± 0.1	2.1 ± 0.1	2.7 ± 0.1
Total P (%)	0.6 ± 0	1.1 ± 0.2	2.2 ± 0.1
Total K (%)	1.2 ± 0.1	1.7 ± 0.1	7.3 ± 0.2
Usage			
Amount (g)	2.1	2.8	16
Additional N fertilization (g_UREA_)	–	–	0.13
Additional K fertilization (g_K2SO4_)	0.15	0.11	0.22

These BBFs were selected due to the high content of both total solids (TS) and organic matter (OM) (Table 1). BBF1 and BBF2 may be considered N organic fertilizers, thanks to their high OM and N contents (Table 1). BBF1 was further characterized by low OM biological stability, as suggested by Adani et al.^17^. The OM of BBF2 was well stabilized due to the process of anaerobic digestion, as explained by Pigoli et al.^18^. BBF3 was characterized by a high content of biologically inert carbon (char) and a high concentration of N, P and K under non-available forms (i.e. oxides)^19^; BBF3 was therefore considered an organic amendment.

In the frame of the FERTIMANURE project, which also aimed at increasing the BBFs’ values, the three BBFs chosen were further activated with the addition of customised microbial consortia to propose a new type of fertilizers: the “activated biofertilizers”.

### Microbial consortia

Three microbial consortia were developed and supplied by AGRIFUTUR Srl (Alfianello (BS), Italy), partner of the FERTIMANURE project, with three different combinations of four bacterial species: (*Azospirillum brasilense*^20^, *Ensifer meliloti*^21^, *Lactiplantibacillus plantarum*^22^ and *Rhizobium pisi*^23^), and three fungal species (*Trichoderma longibrachiatum*^24^ and *T*. *harzianum*^24^ and *Rhizophagus irregularis*^25^) (Table 2). Consortia were made up as follows: Consortium A1—*A. brasilense*, *T. longibrachiatum* and *T*. *harzianum, R. irregularis*, and *L. plantarum*; Consortium A2—*R. pisi, E. meliloti, L. plantarum* and *T. longibrachiatum* and *T. harzianum*; and Consortium A3—*T. longibrachiatum, T*. *harzianum* and*, R. irregularis*.

**Table 2:** Microbial consortia.

BBF	Microorganism	Functional group	Concentration
Microbial consortium 1 (A1)			
BBF1	*Azospirillum brasilense* (mix of three strains)	N-fixer and PGPR	1 g—10^9^ CFU
	*Trichoderma longibrachiatum* and* harzianum*	SOM-degrader	0.25 g—2.5 × 10^8^ spores
	*Rhizophagus irregularis*	P-use efficiency	0.5 g—500 spores
	*Lactiplantibacillus plantarum*	Beneficial effects (PGPR)	0.25 g—50 × 10^9^ CFU
Microbial consortium 2 (A2)			
BBF2	*Rhizobium pisi*	Beneficial effects (PGPR)	1 g—10^9^ CFU
	*Ensifer meliloti* (mix of two strains)	N-fixer and beneficial effects (PGPR)	1 g—10^9^ CFU
	*Lactiplantibacillus plantarum*	Beneficial effects (PGPR)	0.25 g—5 × 10^10^ CFU
	*Trichoderma longibrachiatum* and* harzianum*	SOM-degrader	0.25 g—2.5 × 10^8^ spores
Microbial consortium 3 (A3)			
BBF3	*Trichoderma longibrachiatum* and* harzianum*	SOM-degrader	0.25 g—2.5 × 10^8^ spores
	*Rhizophagus irregularis*	P-use efficiency	0.5 g—500 spores

All these species were selected due to their beneficial effect on plant growth (i.e. rhizobacteria (PGPR), soil organic matter (SOM) degrader, N-fixer and P-use efficiency improvers were selected). Microorganisms linked to SOM mineralisation activity (i.e., *A. brasilense*, *T. longibrachiatum* and *T. harzianum*) were associated with organic BBF1 and BBF2 to enhance N and P availability for plants and to promote OM degradation. Rather differently, BBF3 was associated mainly to microorganisms facilitating P use efficiency (i.e., *R. irregularis, T. longibrachiatum* and *T. harzianum*) as biochar has a high content of poorly available P and furthermore it was inoculated with microorganisms that do not require the presence of degradable SOM to be active. Other microorganisms were selected for their PGPR activities (e.g. *E. meliloti, R. pisi*, *L. plantarum*). The microorganisms were formulated in different ways (information protected) depending on their nature. The final appearance, however, always turned out to be a fine powder of varying nature, depending on the microorganism, so that they could be easily mixed with the BBFs at the time of use.

### Experimental set-up

A greenhouse pot experiment was carried out for 110 days using tomato (*Solanum lycopersicum*^26^ variety Minuet) seedlings as test species. During the experiment, the optimal photoperiod (16 h light and 8 h dark per day) and a temperature of 25 °C were maintained. BBF1 and BBF2 were applied as N organic fertilizers, considering the N requirement of tomato (i.e. complete substitute for mineral fertilizers) and providing the K requirement through the addition of K_2_SO_4_, while BBF3 was applied as an organic amendment providing the N and K requirement through the addition of urea and K_2_SO_4_ respectively, thus supplying all nutrients as for BBF1 and BBF2 (Table 1).

Pots were filled with 2 kg of an agricultural loam soil (Italy) (Table S1) and they were watered to obtain 60% of water holding capacity (WHC) that was maintained throughout the experiment by weighing the pots daily and adding the amount of water transpired/evaporated. Subsequently, fertilizers were applied following an eight treatments scheme: (1) BBF1; (2) BBF1 activated with consortium A1 (ABBF1); (3) BBF2; (4) BBF2 activated with consortium A2 (ABBF2); (5) BBF3; (6) BBF3 activated with consortium A3 (ABBF3); (7) Chemical fertilizer (FC) (0.13 g_UREA_ and 0.22 g_K2SO4_; P was not added due to the high presence of available P in the tested soil (Table S1); 8) Negative control (NC) (no additions). Each treatment had four replicates.

Microbial consortia were added to the BBF just before the experiment was set up, at the dosage indicated in Table 2.

### Yields, quality and nutritional parameters

Tomatoes were collected throughout the crop-season at full ripeness using a colorimeter (Chroma Meter CR-410, Konica-Minolta, Milan, Italy) with the colour parameter set at 25 ± 2 for redness and at 40 ± 2 for darkness^27^. Once harvested, tomatoes were weighed to determine the fresh weight (FW), and then stored at − 20 °C until the end of the experiment keeping the tomatoes produced from each plant segregated (bulk samples). Once the experiment was ended, the bulk samples were homogenised and characterised for total sugar, titrable acidity, sugar:acid ratio, lycopene and carotenoids, as reported in^28^, as well as for taste index^29^ and protein content^30^. At the end of the trial, plants were collected and both fresh and dry (DW) weight (dried at 45 °C until constant weight) registered.

Macro and microelement concentrations of tomatoes, i.e. Na, Mg, K, Ca, P, Mn, Fe, Cu, Zn, Cr, Co, Ni, As, Se, Mo, Cd, Pb were determined by Inductively Coupled Plasma-Mass Spectrometry (ICP-MS, Aurora M90 BRUKER), preceded by microwave assisted (Multiwave ECO, Anton Paar GmbH) nitric acid digestion of dried samples^31^.

Soil characterization was performed according to standard procedures, as described by Pansu and Gauthevrou^32^. BBFs’ characterisation was carried out according to standard procedures of the American Public Health Association and analytical methods for wastewater sludges^33^. The total P and total K in BBFs and the microelement and heavy metal concentrations in soil were again determined by Inductively Coupled Plasma-Mass Spectrometry preceded by microwave assisted nitric acid digestion of dried samples^31^. pH was assessed through a pH meter (Eutech PC 2700, Accuracy: ± 0.002 pH + 1 LSD).

### 16S and ITS rRNA sequencing

Samples for molecular biology analyses were destructively samples at the beginning (Time 0; n = 1), middle (Time 1; n = 1) and end (Time 2; n = 3, pooled sample) of the experimental period. To recover the microbial communities from the rhizosphere, roots were vigorously hand-shaken and the soil which remained attached to the root system was collected^34^. Samples were further homogenized with sterile 2 mm sieves. All samples were stored at − 80 °C prior to DNA extraction.

DNA was extracted in triplicate using a DNeasy PowerSoil Pro Kit (Qiagen, Germany) according to manufacturer’s guidance. Yield and purity of the extracted DNA were quantified on a Nanodrop 1000 spectrophotometer (Thermo Fisher Scientific) while eventual fragmentation was determined through gel electrophoresis 1% (w/v) 1 × TAE agarose gels. Replicates were pulled together to minimize extraction variability. DNA was stored at − 80 °C until analysis.

The NGS was performed at Stab Vida Lda (Lisbon, Portugal). Sequencing targeted the V3 and V4 regions of the bacterial 16S rRNA gene using primers 341F (CCTACGGGNGGCWGCAG) and 785R (GACTACHVGGGTATCTAATCC)^35^ while for the ITS1 regions primers used were ITS1f. (CTTGGTCATTTAGAGGAAGTAA)^36^ and ITS2 (GCTGCGTTCTTCATCGATGC)^37^. The generated DNA libraries were sequenced with MiSeq Reagent Kit Nano in the lllumina MiSeq platform, using 300bp paired-end sequencing reads. The nucleotide sequences generated and analyzed are available at the NCBI SRA repository (BioProject accession number: PRJNA980956). The sequences resulting from the NGS were quality checked through the FastQC software and analyzed using DADA2 for R which was used as per https://benjjneb.github.io/dada2/tutorial.html. Reads were truncated for all analyses at 280 (forward) and 220 (reverse) in order to remove the low-quality section of the reads. The adapter sequence was further removed with the trimLeft function set at the length of the primers for both forward and reverse reads. For taxonomic assignment, the SILVA and UNITE databases were used.

All statistical analyses were performed on R studio (version 4.1.2) as by^31^. Briefly, the package vegan was mainly used^38^ while taxonomic summaries were performed using the phyloseq package^39^. Richness and diversity indexes were calculated and following a Shapiro–Wilk test to test normality, differences among samples of normally distributed data were tested by one-way analysis of variance (ANOVA), followed by a Tukey's post hoc test, while non-normal data were analysed through a non-parametric Kruskal–Wallis test followed by Dunn's Test for multiple comparisons. For pairwise comparison, T-test and Wilcoxon signed-rank tests were used for normal and non-normal data respectively. Beta diversity was tested through a nonmetric multidimensional scaling (NMDS) based on Bray–Curtis distances and then results were confirmed through a PERMANOVA test and pairwise comparisons with the package ‘pairwiseAdonis’.

### Ethical approval

The use of plants in the present study complies with international, national and/or institutional guidelines.

## Results and discussion

### Biomass production and tomato fruit yield

Tomato plant biomass and fruit yields (both FW and DW) achieved at the end of the experiments are reported in Table 3. BBF1 and BBF2, both activated and non-activated, showed similar FW and DW of plant biomass with respect to NC, while being significantly lower, as expected, than the FC (Table 3). Conversely, BBF3 and ABBF3 showed the highest biomass production, for both FW and DW, across all treatments, even higher than the FC. Interestingly, no differences between non-activated and activated BBFs were observed for plant biomass production. When considering tomato fruit biomass, the yield decreased following the order: ABBF3 > ABBF2 > BBF3 > FC > ABBF1 > BBF2 > BBF1 > NC. In general, all ABBFs showed higher tomato weight than the non-activated BBFs, and were mostly similar to (ABBF1) or higher (ABBF2 and ABBF3) than FC (Table 3). As expected, BBF3 showed a tomato fruit yield similar to that of FC since the pots received the same synthetic fertilizer application (BBF3 was used as an organic amendment). Conversely, BBF1 and BBF2 (organic-N fertilizers) that were used as substitute of mineral fertilizers showed a lower production than FC, and this was expected since the nutrient replacement value of organic fertilizers is lower than those of chemical fertilizers. An organic N-form requires time to be mineralized into readily plant available forms, resulting in a non optimal synchronization of organic N mineralization and crop N demand^40,41^. Nevertheless, inoculation with microbial consortia was able to overcome the low nutrient availability typical of organic fertilizers in ABBF1 and even more in ABBF2, achieving fruit production values, respectively, similar or higher when compared to FC (Table 3). This was in accordance with^42^, in which a microbial inoculum added to organic fertilizers not only increased the assimilation of nutrients (N, P, and K) in maize plants, but also resulted in the highest biomass and seedling height.

**Table 3:** Productive yields of tomato plants (plant and tomato fruits) in the pot experiment (average ± standard deviation (n = 3)). Letters indicate statistically different weights across treatments according to Tukey test (*p* ≤ 0.05).

	NC	FC	BBF1	ABBF1	BBF2	ABBF2	BBF3	ABBF3
Plant biomass								
Fresh weight (g)	20.2 ± 3.3a	30.4 ± 2.8b	18.4 ± 2.8a	21.4 ± 2.2a	20 ± 1.2a	23.3 ± 3a	49 ± 2.7c	49.9 ± 4.4c
Dried weight (g)	9.1 ± 1.5a	13.1 ± 1.2b	8.1 ± 0.6a	10.3 ± 1a	8.6 ± 0.5a	9.8 ± 1.3a	24.5 ± 1.4c	25 ± 2.2c
Tomato fruit biomass								
Fresh weight (g)	52.6 ± 7.6a	107.9 ± 3.8c	88.2 ± 8.4b	101.6 ± 3.9c	89 ± 5.5b	120.5 ± 2.4d	110.5 ± 6.9b	127 ± 4.8d
Dried weight (g)	5.8 ± 1.1a	12.1 ± 0.9c	9.4 ± 0.9b	11.7 ± 0.7c	7.9 ± 1.4b	13.1 ± 0.5d	11.8 ± 0.3b	14.1 ± 2.1d

Concerning BBF1, the lower productions of biomass and tomato fruits observed with respect to FC were probably ascribable also to the low biological stability of bio-dried materials which exercised phytotoxicity activity, as indicated by the presence of plant phytotoxicity symptoms registered above all at the early stage of the trial (i.e. yellowish leaves, reduced growth)^43^. This could be expected, since bio-dried materials do not undergo a complete stabilization process (i.e. composting), and this results in the presence of easily biodegradable organic compounds in the product that can lead to detrimental effects in soil and to phytotoxicity^43^. Nevertheless, the inoculation of BBF1 with microbial consortia resulted in mitigation of the phytotoxicity, to allow the obtaining of the positive results of ABBF1 previously discussed (Table 3).

The different pattern shown by BBF3, i.e. high biomass production for both BBF and ABBF3, was probably due to the fact that it was used as an organic amendment together with the addition of complete mineral fertilizers. In this case, biochar may have led to a more efficient nutrient availability thanks to its numerous positive effects (i.e. high water retention, high exchange capacity etc.)^44,45^. This advantage deriving from biochar application can be found in the literature, confirming our results, for example, de la Rosa et al.^46^ and Schimmelpfennig et al.^47^ who reported an increased biomass production of up to + 300% and + 50% after application of biochar to grasslands. In a greenhouse experiment on tomato plants, Calcan et al.^48^ achieved 50% higher values of height, number of leaves, and collar diameter of plants when biochar was added. In the present experiment, inoculation of BBF3 (ABBF3) was characterised by an even higher production than FC, probably due to an increased mobilization of nutrients and the biostimulant effects of the microorganisms applied.

The activation of BBFs with ad hoc microbial consortia led to an enhanced total productivity of tomato fruits in all the trials, overcoming phytotoxicity and scarce nutrients bioavailability concerns of BBFs. This further supports the thesis that, similarly to what has been reported with synthetic fertilizers^49^, microbial activation is an efficient technique to increase nutrient availability. It is therefore of the utmost importance to follow the less pursued strategy and design new fertilizers in combination with ad hoc consortia to improve the yields deriving from organic fertilizer usage and to substitute synthetic fertilizers and dependence on primary raw materials^50^.

### Quality of tomato fruits and nutritional value

The quality and nutritional value of tomatoes produced in the greenhouse experiment were also evaluated. To do that, total solids (TS), total soluble sugars (TSS), titrable acidity (TA), sugar:acid ratio (TSS/TA), pH, taste index (TI), protein, carotenoids and lycopene contents were measured (Table 4). TS, TA, and pH did not show any significant differences between treatments.

**Table 4:** Quality and nutritional parameters of tomato fruits. (average ± standard deviation (n = 3)). Letters indicate statistically different weights across treatments according to Tukey test (*P* ≤ 0.05). Data are expressed on fresh weight basis except for * that are expressed on dry weight basis.

	NC	FC	BBF1	ABBF1	BBF2	ABBF2	BBF3	ABBF3
Total solids (%)	12.6 ± 1.3	10.1 ± 1.2	9.2 ± 0.3	10.6 ± 0.5	9.5 ± 0.8	10.2 ± 0.3	10.4 ± 0.8	12.4 ± 1
Total soluble sugars (°Brix)	6.3 ± 0d	7.1 ± 0b	6.7 ± 0.1c	6.8 ± 0c	6.7 ± 0.1c	6.9 ± 0.1c	7.3 ± 0.1a	7.2 ± 0.1ab
Titrable acidity (% Citric Acid)	0.56 ± 0.01	0.52 ± 0.04	0.55 ± 0.02	0.55 ± 0.02	0.61 ± 0.06	0.57 ± 0.03	0.67 ± 0.01	0.62 ± 0
Sugar:Acid ratio	11.2 ± 0.3d	13.8 ± 1.2a	12.2 ± 0.4b	12.3 ± 0.4b	11 ± 0.1d	12 ± 0.1b	11 ± 0.1d	11.7 ± 0.1c
pH (pH unit)	4.13 ± 0.05	4.12 ± 0.02	4.18 ± 0.02	4.16 ± 0.01	4.12 ± 0.03	4.1 ± 0.05	4.1 ± 0.01	4.05 ± 0.02
Taste Index	1.12 ± 0c	1.21 ± 0.02a	1.16 ± 0b	1.16 ± 0b	1.16 ± 0b	1.17 ± 0.01b	1.22 ± 0a	1.2 ± 0a
Protein* (%)	3.26 ± 0.23d	4.07 ± 0.08b	4.25 ± 0.18a	3.98 ± 0.12b	4.44 ± 0.1a	4.4 ± 0.21a	4 ± 0.07b	3.64 ± 0.1c
Carotenoids (µg g^−1^)	32.8 ± 2.9d	60.2 ± 5.7c	63.6 ± 11.9c	97.5 ± 7.7b	55.9 ± 12.0c	82.7 ± 7.2c	99.9 ± 4.9b	129.4 ± 7.4a
Lycopene (µg g^−1^)	25.6 ± 2.3d	46.9 ± 4.4c	49.6 ± 9.3c	76 ± 6b	50.3 ± 4.1c	64.5 ± 7.2c	78 ± 4.9b	101 ± 7.3a
K* (µg g^−1^)	26,283 ± 46	25,904 ± 1415	28,864 ± 2052	28,985 ± 724	28,369 ± 2675	26,400 ± 1669	31,359 ± 910	30,413 ± 2475
P* (µg g^−1^)	3937 ± 1	3701 ± 154	4106 ± 171	4183 ± 107	4059 ± 346	3900 ± 284	4358 ± 104	4256 ± 266
Ca* (µg g^−1^)	776 ± 15bc	997 ± 102ab	744 ± 75bc	694 ± 42c	838 ± 69bc	1141 ± 12a	895 ± 45abc	987 ± 130ab
Mg* (µg g^−1^)	1293 ± 5ab	1225 ± 84b	1451 ± 20ab	1453 ± 29ab	1345 ± 95ab	1352 ± 98ab	1410 ± 89ab	1518 ± 73a
Na* (µg g^−1^)	223 ± 15c	266 ± 26 bc	266 ± 32bc	287 ± 4 bc	236 ± 21 c	340 ± 23 b	536 ± 15 a	596 ± 47a
Fe* (µg g^−1^)	25.4 ± 3.4bc	23.6 ± 1.9c	22.3 ± 2.5c	22.3 ± 0.8c	24.8 ± 3.8bc	27.4 ± 1.5a	32.5 ± 0.2b	28.9 ± 2bc
Al* (µg g^−1^)	32.45 ± 2.29	35.25 ± 4.92	32.45 ± 4.59	27.46 ± 15.62	9.02 ± 3.38	15.07 ± 0.53	4.29 ± 0.57	7.83 ± 1.01
Cu* (µg g^−1^)	7.61 ± 0.61b	6.76 ± 0.08b	5.48 ± 0.34b	6.2 ± 0.92b	5.5 ± 0.29b	5.89 ± 0.01b	7.51 ± 0.37b	10.26 ± 0.99a
Zn* (µg g^−1^)	15.85 ± 3.82	17.66 ± 6.51	15.58 ± 5.52	17.44 ± 1.91	13.21 ± 1.36	29.12 ± 8.14	17.28 ± 3.65	19.65 ± 0.23

TSS concentration seemed to be related to the application of fertilizers, both chemical and organic, since there were small differences between all the treated plants (i.e. ranging from 6.7 to 7.3°Brix), but all showed a higher TSS concentration than control (NC), except BBF2 and BBF3 that had a TSS/TA similar to NC. These results were in accordance with Hernández et al.^51^ who reported that quality parameters of tomato fruit did not differ significantly when plants were treated with inorganic or organic fertilizers. Overall, for the parameters related to the organoleptic quality of tomato fruits (i.e. TSS, TA, TSS/TA, TI), the effect of microorganisms’ inoculation was negligible or almost negligible if compared to the application of the non-activated BBFs.

Considering nutritional values, all the treated tomato fruits showed a higher concentration of protein with respect to the NC, ranging from 3.6 ± 0.1% w/w (ABBF3) to 4.4 ± 0.1% w/w (BBF2), and this might be related to the N fertilization applied that allowed for an increased N accumulation in the fruits^52^. As reported for the organoleptic parameters, also for the proteins, there were no clear differences among fertilized treatments. Conversely, a clear benefit of microbial inoculation to BBFs was evident for carotenoids and lycopene content. Considering FC as a baseline, carotenoid content changed by about + 6%, − 7% and 66% for BBF1, BBF2 and BBF3, respectively, while lycopene content increased by about + 6%, + 7% and + 66% for BBF1, BBF2 and BBF3, respectively (Table 4). A further increase was seen with microbial inoculation. Considering BBFs vs. ABBFs, microbial activation induced an increase in carotenoid content of about + 53%, + 48 and + 30 for ABBF1, ABBF2 and ABBF3, respectively, while lycopene content increased by about + 53%, + 28% and + 29% for ABBF1, ABBF2 and ABBF3, respectively (Table 4). Carotenoids and lycopene are the most characteristic antioxidant compounds of tomato, and they are responsible of the colour of the ripe fruit. Their intake has been also correlated to a reduced risk of certain types of cancer and cardiovascular disease^29^. An increased content of carotenoids and lycopene in tomato fruits, following the fertilization with ABBFs represents an interesting result that needs to be further studied to understand the metabolic reasons behind it. For example, *Trichoderma* (one of the fungal components of the ABBFs) seems to drive sugar accumulations which are then used by the plant for the production of components such as carotenoids^53^. Similarly, other pathways might be enhanced or inhibited by the other members of the consortia.

Additionally, an ionomic analysis was carried out (Table 4 and Table S2). When considering Na, all samples showed similar values except for BBF3 and ABBF3 which showed a higher accumulation (+ 101% and + 124% respectively than FC) and ABBF2 which was characterised by a significant increase when compared to BBF2 (+ 44%). Calcium was also characterised by a trend of higher accumulation led by microbial activation in BBF2 and BBF3 while BBF1 showed the opposite trend, however values remained similar to or slightly lower than FC. For both Mg and Cu the highest value was retrieved in ABBF3.

To sum up, quality and nutritional value assessment of tomato fruits showed that microbial activation of BBFs did not affect organoleptic parameters, but it enhanced carotenoids and lycopene concentration, providing an added nutritional value to the fruits.

### Bacterial communities

NGS analyses of the rhizospheric soil collected from all the different treatments, allowed us to monitor the persistence of the microorganisms of the three consortia across time. At a phylum level Consortium 1 (A1) and 2 (A2) were characterised by a dominance of Firmicutes (now Bacillota) 99.4% and 93.4%, respectively with a small presence (6.6%) of Proteobacteria in A2 (Figure S1). This is partially expected as *Lactobacillus plantarum* belongs to Firmicutes while all other microorganisms *Rhizobium pisi, Sinorhizobium meliloti and Azospirillum brasilense* belong to Proteobacteria. The high abundance of *Lactobacillus plantarum* was probably due to its higher concentration. Consortium 3 (A3) is vastly different, since it is a combination of two fungal inocula (*Trichoderma spp.* and *Rhizophagus irregularis*). These samples were characterised by a high abundance of Proteobacteria (22.3–6.6%), Planctomycetota (21.8–9.6%) and Acidobacteria (16.2–9.1%) followed by other phyla.

At a genus level, as expected, A1 was characterised by a dominance of *Lactiplantibacillus plantarum* (99.4%) with only traces (> 2%) of other bacteria and especially of *Azospirillum brasilense* (0.05%). When considering A2, the dominant genus was again *L. plantarum* (93.3%) followed by *Ensifer meliloti* (3.9%), *Rhizobium pisi* (2.2%) as expected. When looking at A3 the composition was variable and dominated by species of low abundance, among which *L. plantarum* (0.4%) and *Azospirillum* (1.8%) were retrieved.

In terms of beta diversity, a shift of the communities at Time 2 can be seen, however, no pattern across treatments was identified (Figure S1). When looking at permanova analysis, time was a variable significantly affecting the bacterial community (*p* = 0.001) with both Time 0 and Time 1 again differing from Time 2 while fertilizers and the amendments did not seem to have a significant influence (Figure S1).

**Figure 1:**
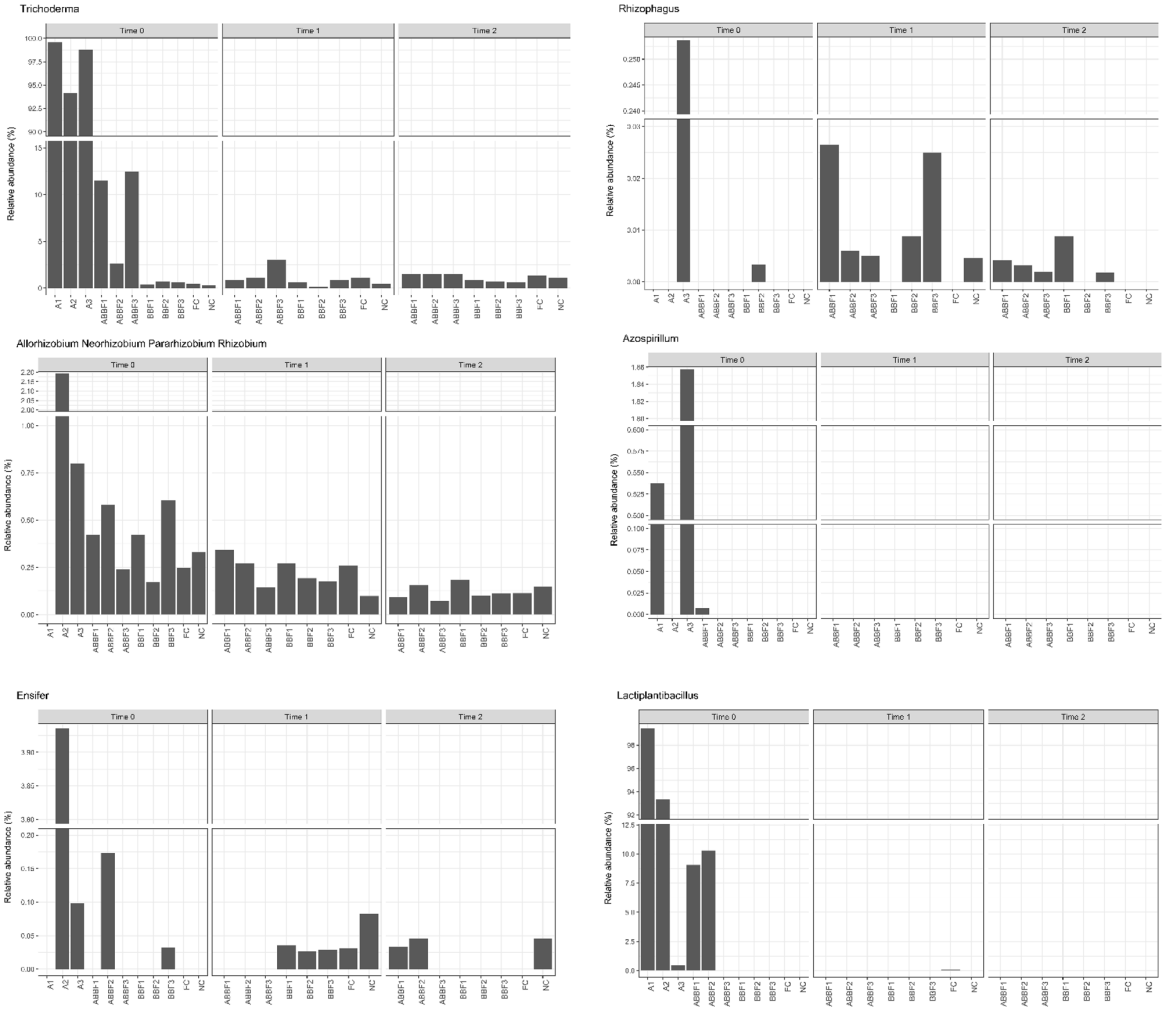
Bar-plots of the consortia microorganisms across treatments and time.

When looking at the permanence of these inocula across time within the rhizospheric soil (Fig. 1), it can be seen that *L. plantarum* was only retrieved in the amended pots at Time 0 (ABBF1: 9.1%, ABBF2: 10.3% and ABBF3: 0.02%) and in the soil treated with FC at Time 1 (0.1%). *A. brasilense* was retrieved only in ABBF1 at Time 0 (0.01%) but not at later sampling. At time 0, *E. meliloti* was present as expected in ABBF2 (0.17%) but also in the BBF3 (0.03%). When looking at time 1, *E. meliloti* was only present in samples not amended (NC: 0.08%, FC: 0.03%, BBF1: 004%, BBF2: 0.03%, BBF3: 0.03%). While when looking at Time 2, *E. meliloti* was again present in NC (0.05%) and in both ABBF1 (0.03%) and ABBF2 (0.05%). Amplicon sequence variants for *E. meliloti* differed however between the ones retrieved at Time 0 and the ones retrieved at Time 1 and 2; it can be therefore suggested that the amplicon sequence variants (ASVs) retrieved after Time 0 might be different species and not be connected to the inoculum. *R. pisi* was ubiquitous to all samples at all times with an abundance ranging from 0.07 and 0.6% with a general trend of decrease across time. No statistical differences were retrieved between amended or not amended samples.

In terms of alpha diversity, no main differences were identified, however a trend of lower richness and diversity seemed to characterise NC when compared to all other samples, FC included (Table S3).

The added bacteria were selected for their stimulating characteristics which are commonly known across literature but still require a more in-depth characterisation, especially to understand their interaction with bio-based fertilizers. *L. plantarum*, for example, is one of the most versatile and metabolically diverse *Lactobacillus*. *L. plantarum* has been widely used in industrial fermentation and has been isolated from various sources, however, rarely from rhizospheric soil^54^. Having a generally recognized as safe (GRAS) status, *Lactobacillus* and *Lactiplantibacillus* can be used for agronomically relevant plants for human consumption without harmful effects on human health. In combination with other microorganisms, *L. plantarum* enhances decomposition of organic materials leading to a quicker and better release of nutrients^55^. When applied directly to tomato seeds, it showed PGPR activity leading to an increase in germination, shoots length, number of main and lateral roots and root hairs^56^. Effects seem to be strain specific: however, studies are still lacking. Some studies also indicate possible biocontrol agent activities^57^. *A. brasilense*, a free-living nitrogen-fixing bacterium, was selected for its status as a model bacterium for plant growth promotion and free-living nitrogen fixation. Commonly found in rhizospheric soil, *A. brasilense* has been used as a bio-inoculant for cereal crops, in which its presence led to an increase in the number of lateral roots, root hairs, shoot mass and internal levels of auxin, with further changes also detected in the root transcriptome^58^. On tomato plants, *A. brasilense* (strain Sp. 7) application led to increased above-ground biomass of tomato seedlings, probably due to nitrogen-fixation^59^. On the other hand, *E. meliloti* and *R. pisi* are both legume nodulating bacteria (LNB) that can be found both in symbiotic or free-living conditions. *R. pisi* is a broad bean rhizobium. *Rhizobium* often leads to a higher tolerance to environmental stresses such as low soil pH, high temperatures and antimicrobial presence ^60^. Additionally, *Rhizobium* species have been shown to have endosymbiotic relationships with plants outside their normal association and also to stimulate plants outside the symbiosis, therefore it is relevant to assess their effects in non-canonic interactions^61^. *E. meliloti* is a free-living bacterium that performs N fixation when it differentiates into an endosymbiotic Bacteroide of alfalfa as a result of a spectrum of phenotypic variations in the plant-rhizobium relation due to genetic determinants from both sides^62^. Plant growth-promoting rhizobacteria (PGPR) have been isolated from legume nodules, displaying a potential to enhance nodulation, growth and yield of legume plants when co-inoculated with Rhizobium. No studies were found on the effect of *E. meliloti* and *R. pisi* on tomato plants.

From the results obtained (i.e. presence of the bacteria only at Time 0 or retrieved after Time 0 without a specific pattern) it seemed that these bacteria might not be effective or were effective only at the early stage. After Time 0, their survival might be hindered by the soil and rhizospheric communities or reduced below detectable limits. Another reason for their low retrieval might be that the fertilization technique, that is mimicking an on-field spreading, might not be as suitable for these bacteria as it might be for their fungal “partners”. Commonly known techniques to enhance their efficiency might be seed coating or seedling’s roots dipping. However, new techniques need to be developed to use microbial activation on-field especially in combination with BBFs’ application^15^.

### Fungal communities

An NGS analysis was also carried out to identify and understand the permanence of the fungal microorganisms within the rhizospheric soil. In terms of phylum, all rhizospheric samples showed similar fungal composition with a majority of Ascomycota and Mortierellomycota and a minor presence of Basidiomycota and sporadically of Chytridiomycota (Figure S1).

When considering the consortia, at a genus level, as expected, A1 was characterised by a dominance of *Trichoderma* spp*.* (a mix between *T. longibrachiatum* and *T. harzianum*) (99.6%) followed by other fungi at a lower abundance; similarly, A2 and A3 again showed a predominance of *Trichoderma,* 94.2% and 98.7% respectively. *Rhizophagus* spp. (*diaphanus*) was only retrieved at low abundance (0.003%) in A3. Additionally, an unknown Glomeraceae was present in both A1 and A3 although at low abundance (0.0004 and 0.003%, respectively).

Similarly to bacterial communities, the beta-diversity of fungal communities was significantly affected by time (*p* < 0.01) across all rhizospheric samples, with Time 2 being dissimilar from Time 1 and Time 0 (Figure S1).

When looking at the permanence of these inocula across time in the rhizospheric soil (Fig. 1), *Trichoderma* spp*.* were present in all samples, across time they showed a trend of higher abundance within amended samples with a significant difference at Time 2 (*p* < 0.001) (Time 0—BBFs: av. 0.6%, ABBFs: 8.8%; Time 1—BBFs: av. 0.5%, ABBFs: 1.7%; Time 2—BBFs: av. 0.7%, ABBFs: 1.5%). Other species of *Trichoderma* were also retrieved in samples at high abundances (e.g. *T. theobromicola* and *T. virens*). *Rhizophagus* was present in most samples with an abundance up to 0.2% with no particular trend.

*Trichoderma* genus is a group of anamorphic filamentous fungi, ubiquitously found in the presence of cellulosic materials, in particular in the rhizosphere. *Trichoderma* are plant symbionts which colonize the outer layers of roots without entering the vascular bundle. Their symbiotic abilities, coupled with a high adaptability to various environments and a fast growth rate, benefit *Trichoderma* over other filamentous fungi^63^. Currently used in the production of industrial bio-based fertilizers, *Trichoderma* spp*.* are characterised by a range of advantageous effects on crop production, i.e., improved productivity and nutritional quality, pathogens/pests biocontrol and resistance to numerous environmental stresses^64^. *Trichoderma* is therefore defined as a “booster” for faster and healthier growth. *Trichoderma* acts on a multilevel root–shoot communication, influencing plants both directly and indirectly by releasing (1) substances with auxin-like activity, (2) small peptides, (3) volatile organic compounds; therefore, leading to a general improvement in root architecture and in the assimilation or solubilization of both macronutrients leading to the higher yield and quality^10^. Additionally, *Trichoderma* are able to interact with other rhizospheric microorganisms, therefore affecting, both quantitatively and qualitatively, microbial populations^65^. Many species are capable of establishing an endophytic mutualistic relationship with different plants. Inoculation of *T. longibrachiatum*, a species with little studied potential in agriculture, on tomato plants, resulted in significant biostimulation effects on plant growth and improved recovery after stress^66^. *T. longibrachiatum* is also known to produce antifungal molecules which can suppress tomato gray mould and tomato late blight^67^. Similarly, *T. harzianum* showed its importance as a biocontrol agent on tomato plants. Similarly to this study ^53^, found that tomato inoculation with *T. harzianum* strain T22 led to higher accumulation of sugars which in turn enhanced the production of nutrients, hormones and metabolites. In this context, the higher production glycolytic enzymes increased the C supply to biosynthetic pathways enhancing the production of plant resistance-secondary metabolites such as carotenoids.

*Rhizophagus* are arbuscular mycorrhizal fungi. On tomato plants, *R. irregularis* acted as a biostimulant inducing a lower disease incidence, higher chlorophyll production and higher accumulation of P, K, Zn, Cu and Mo^68^; the effect of *R. diaphanus* on tomato plants is still to be explored.

Summarizing, when compared to bacterial inoculants, fungal inoculants showed a higher permanence in soil and were also retrieved more frequently after Time 0. *Rhizophagus* was present at low abundance and sporadically which might hint to a “casual presence” probably not correlated to the fertilization. Similarly, *Trichoderma* was also retrieved in all samples leading to the conclusion that *Trichoderma* is already a component of the soil community under study. However, when “spiked” its presence was higher across time in the ABBFs, leading to the conclusion that *Trichoderma* is playing the leading role out of all the microorganisms and driving the biomass and nutritional differences. The fertilization technique used seems to be better suited to fungi, for which it is less problematic than for their bacterial counterparts.

### Benefits of microbially activated bio-based fertilizers

Biological activated BBFs showed high performance for tomato plants fertilization, resulting in increased fruit yields and quality (i.e. lycopene and carotenoids concentration) with respect to non-fertilized and chemically fertilized plants. Although the results presented in this study need further confirmation, they may have interesting implications in the context of Circular Economy Strategy for Europe and of Sustainable Development Goals (SDGs) as defined by the United Nations ^69,70^.

Biological activation of BBFs may help overcome the drawbacks of BBFs that still limit their spread in agriculture. The faster mobilization of nutrients (i.e. N and P) and the overall biostimulant effects have been proved to enhance the fertilizer value of BBFs, making them a feasible and complete substitute for synthetic fertilizers. The reclamation of organic matter and nutrients from animal manures in agricultural soils through application of biological activated BBFs may reduce the dependence on synthetic fertilizers, with clear economic and environmental consequences. Economically, reduction in the use of synthetic fertilizers (e.g., urea, potassium chloride and diammonium phosphate) is becoming mandatory for farmers to sustain global food production cost-effectively^71^. Through the lowering of animal manure disposal costs and the production of high-added value biofertilizers, transforming animal manures in biological activated BBF with a high fertilization value may also reduce the economic costs of livestock production and introduce new incomes for farmers. From an environmental perspective, the application of biologically activated BBFs as replacements for synthetic fertilizers may have two important implications. In addition to the reduction of greenhouse gas emissions into the atmosphere, and the leaching into groundwaters caused by synthetic fertilizers application to the soil, the reduction of dependence on non-renewable fossil raw materials whose extraction and transformation has a high environmental impact must also be considered. Overall, biological activation of BBFs is clearly meeting the principles of the Circular Economy Strategy, which aims to encourage a more sustainable and effective use of resources by minimising waste, increasing recycling, and minimising the extraction of raw materials^72^.

Enhancing food production and quality through a Circular Economy approach may be also useful in achieving several of the sustainable development goals (SDGs) defined in 2015 by United Nations (e.g., SDG2 (No hunger), SDG11 (Responsible consumption and production), SDG13 (Climate Action), SDG14 (Life below water), and SDG15 (Life on land)). Taking this into account, the need to further study and develop microbial consortia to produce efficient biologically activated BBFs becomes urgent. In future assessments, technical, economic and environmental evaluations are required to provide further evidence of sustainability of agricultural application of biological activated BBFs, both in pot and in field experiments.

## Conclusions

This study evaluated three different BBFs activated with different microbial consortium for their fertilization value in tomato fruit production, as well as for their effects on rhizospheric communities.

Overall, biological activation enhanced fertilization performance of BBFs, and the tomato fruit yields of biologically activated BBFs trials were even higher than the chemically fertilized ones, proving that this approach may completely replace synthetic fertilizers. Tomato fruit quality (i.e. lycopene and carotenoids concentration) was also improved by biological activation of BBFs, indicating that secondary metabolism pathways in plants were affected by the microbial inoculation of BBFs. Molecular biology analysis pointed out that *Trichoderma* was the main driver of the positive effect on tomato yields, whereas bacteria might be not effective or effective only at the early stage.

To stimulate the full-scale application of biological activated BBFs and thus to meet Circular Economy Strategy and SDGs objectives, developing new techniques to use microbial activation on-field, especially in combination with BBFs application, represents the main objective of future studies.

### Supplementary Information

Supplementary Information.

## Data Availability

The datasets generated during and/or analysed during the current study are available in the NCBI SRA repository (BioProject accession number: PRJNA980956) [https://www.ncbi.nlm.nih.gov/bioproject/PRJNA980956/].
