# Supplementary material for: Effects of the application of microbiologically activated bio-based fertilizers derived from manures on tomato plants and their rhizospheric communities
Source: Sci Rep. 2023 Dec 18;13:22478. doi: 10.1038/s41598-023-50166-5 (PMC10728056; doi:10.1038/s41598-023-50166-5)
Supplement: Supplementary file 1 — Supplementary Information. [file 41598_2023_50166_MOESM1_ESM.docx]

**Supplementary materials**

**for**

# Effects of the application of microbiologically activated bio-based fertilizers derived from manure on tomato plants and their rhizospheric communities.

Elisa Clagnan^a,1^, Mirko Cucina^a,b,1^, Patrizia De Nisi^a^, Marta Dell’Orto^a^, Giuliana D’Imporzano^a^, Roberto Kron-Morelli^c^, Laia Llenas-Argelaguet^d^, Fabrizio Adani^a*^.

^a^ Gruppo Ricicla labs., Dipartimento di Scienze Agrarie e Ambientali - Produzione, Territorio, Agroenergia (DiSAA), Università degli studi di Milano, Via Celoria 2, 20133 Milano, Italy.

^b^ National Research Council of Italy, Institute for Agriculture and Forestry Systems in the Mediterranean (ISAFOM-CNR), Via della Madonna Alta 128, 06128 Perugia, Italy

^c^ Agrifutur Srl, Via Campagnole 8, 25020 Alfianello (BS), Italy.

^d^ BETA Tech Center, TECNIO Network, University of Vic-Central University of Catalonia, Ctra de Roda 70, 08500, Vic, Spain.

^1^ Elisa Clagnan and Mirko Cucina contributed equally to this work.

*** Corresponding Author:** Fabrizio.Adani@unimi.i

# Supplementary tables

**Table S1.**  Soil characterisation (average ± standard deviation (n=3)). Data are expressed on dry weight basis except for pH.

|  | **Soil** |
| --- | --- |
| **pH** (pH unit) | 6.8 ± 0.2 |
| **Total organic C** (%) | 1.61 ± 0.09 |
| **Organic matter** (%) | 2.77 ± 0.15 |
| **Total N** (%) | 0.11 ± 0.01 |
| **C/N** | 14.6 |
| **Available P_2_O_5_** (mg kg^-1^) | 110 ± 6 |
| **Exchangeable K_2_O** (mg kg^-1^) | 360 ± 24 |
| **Ni** (mg kg^-1^) | 18 ± 3 |
| **Cu** (mg kg^-1^) | 19 ± 1 |
| **Zn** (mg kg^-1^) | 53 ± 1 |
| **As** (mg kg^-1^) | 14 ± 3 |
| **Cd** (mg kg^-1^) | Below detection limit (< 0.1) |
| **Pb** (mg kg^-1^) | 18 ± 3 |
| **Hg** (mg kg^-1^) | < 0.5 |
| **Total Cr** (mg kg^-1^) | 26 ± 2 |
| **CrVI** (mg kg^-1^) | < 0.2 |
| **Water Holding Capacity** (%) | 30 ± 1 |
| **Cation exchange capacity** (mg kg^-1^) | 22 ± 3 |
| **Sand** (%) | 45 |
| **Silt** (%) | 44 |
| **Clay** (%) | 11 |

**Table S2.** Heavy metals present in tomato fruits. (average ± standard deviation (n = 3)). Letters indicate statistically different weights across treatments according to Tukey test (P ≤ 0.05). Data are expressed on dry weight basis.

|  | **NC** | **FC** | **BBF1** | **ABBF1** | **BBF2** | **ABBF2** | **BBF3** | **ABBF3** |
| --- | --- | --- | --- | --- | --- | --- | --- | --- |
| **Cr** (µg g^-1^) | u.d.l | u.d.l | 0.061 ± 0.012 | 0.065 ± 0.051 | 0.109 ± 0.105 | 0.051 ± 0.031 | u.d.l | 0.157 ± 0.099 |
| **Mn** (µg g^-1^) | 7.471 ± 0.101 | 6.833 ± 0.276 | 8.07 ± 0.176 | 8.216 ± 0.652 | 7.713 ± 0.034 | 7.757 ± 0.37 | 7.365 ± 0.236 | 8.196 ± 0.619 |
| **Co** (µg g^-1^) | u.d.l | u.d.l | u.d.l | u.d.l | u.d.l | u.d.l | u.d.l | u.d.l |
| **Ni** (µg g^-1^) | 0.057 ± 0.013 c | 0.242 ± 0.531 c | 0.185 ± 0.152 c | 0.296 ± 1.101 bc | 0.224 ± 0.404 c | 2.44 ± 0.426 a | 0.087 ± 0.011 c | 1.944 ± 0.488 ab |
| **Mo** (µg g^-1^) | u.d.l | u.d.l | u.d.l | u.d.l | u.d.l | u.d.l | u.d.l | u.d.l |
| **Pb** (µg g^-1^) | u.d.l | 0.027 ± 0 | u.d.l | u.d.l | 1.225 ± 0.613 | 0.412 ± 0.168 | u.d.l | 0.218 ± 0.02 |
| **Se** (µg g^-1^) | 0.123 ± 0.051 | 0.05 ± 0.056 | 0.193 ± 0.111 | 0.014 ± 0 | 0.01 ± 0 | 0.01 ± 0 | u.d.l | 0.344 ± 0.39 |
| **As** (µg g^-1^) | u.d.l | u.d.l | u.d.l | u.d.l | u.d.l | u.d.l | u.d.l | u.d.l |
| **Cd** (µg g^-1^) | 0.073 ± 0.017 | 0.113 ± 0.029 | 0.046 ± 0.021 | 0.082 ± 0.008 | 0.121 ± 0.046 | 0.099 ± 0.015 | 0.058 ± 0 | 0.126 ± 0.014 |

**Table S3.** Observed richness, Shannon and Simpson alpha diversity and evenness indexes for eukaryotic and prokaryotic communities.

|  | **Eukaryote** | | | |  | **Prokaryote** | | | |
| --- | --- | --- | --- | --- | --- | --- | --- | --- | --- |
|  | **Observed richness** | **Shannon diversity index** | **Simpson diversity index** | **Pielou's evenness** |  | **Observed richness** | **Shannon diversity index** | **Simpson diversity index** | **Pielou's evenness** |
| **Time 0** |  |  |  |  |  |  |  |  |  |
| **A1** | 16 | 0.33 | 0.13 | 0.12 |  | 10 | 0.14 | 0.05 | 0.06 |
| **A2** | 13 | 1.00 | 0.54 | 0.39 |  | 18 | 0.41 | 0.16 | 0.14 |
| **A3** | 38 | 0.39 | 0.14 | 0.11 |  | 1203 | 6.17 | 1.00 | 0.87 |
| **NC** | 802 | 4.75 | 0.98 | 0.71 |  | 1370 | 6.68 | 1.00 | 0.92 |
| **FC** | 976 | 5.07 | 0.98 | 0.74 |  | 1712 | 6.83 | 1.00 | 0.92 |
| **BBF1** | 827 | 4.87 | 0.98 | 0.72 |  | 1557 | 6.75 | 1.00 | 0.92 |
| **BBF2** | 1004 | 5.19 | 0.99 | 0.75 |  | 1265 | 6.59 | 1.00 | 0.92 |
| **BBF3** | 915 | 4.98 | 0.98 | 0.73 |  | 1704 | 6.86 | 1.00 | 0.92 |
| **ABBF1** | 903 | 4.80 | 0.98 | 0.71 |  | 2488 | 6.79 | 0.99 | 0.87 |
| **ABBF2** | 980 | 5.11 | 0.98 | 0.74 |  | 1490 | 6.44 | 0.99 | 0.88 |
| **ABBF3** | 1001 | 4.87 | 0.98 | 0.70 |  | 2183 | 7.06 | 1.00 | 0.92 |
| **Time 1** |  |  |  |  |  |  |  |  |  |
| **NC** | 858 | 4.20 | 0.93 | 0.62 |  | 913 | 6.31 | 1.00 | 0.93 |
| **FC** | 912 | 4.85 | 0.98 | 0.71 |  | 1806 | 6.93 | 1.00 | 0.92 |
| **BBF1** | 780 | 4.31 | 0.95 | 0.65 |  | 2083 | 6.98 | 1.00 | 0.91 |
| **BBF2** | 510 | 3.55 | 0.91 | 0.57 |  | 2904 | 7.32 | 1.00 | 0.92 |
| **BBF3** | 810 | 4.31 | 0.94 | 0.64 |  | 2084 | 7.02 | 1.00 | 0.92 |
| **ABBF1** | 871 | 4.65 | 0.97 | 0.69 |  | 1820 | 6.99 | 1.00 | 0.93 |
| **ABBF2** | 1028 | 4.74 | 0.97 | 0.68 |  | 1782 | 6.94 | 1.00 | 0.93 |
| **ABBF3** | 876 | 4.69 | 0.97 | 0.69 |  | 2136 | 7.08 | 1.00 | 0.92 |
| **Time 2** |  |  |  |  |  |  |  |  |  |
| **NC** | 994 | 4.43 | 0.95 | 0.64 |  | 1349 | 6.44 | 0.99 | 0.89 |
| **FC** | 948 | 4.29 | 0.94 | 0.63 |  | 1641 | 6.70 | 1.00 | 0.91 |
| **BBF1** | 1008 | 4.60 | 0.96 | 0.67 |  | 1180 | 6.58 | 1.00 | 0.93 |
| **BBF2** | 926 | 4.51 | 0.96 | 0.66 |  | 1742 | 6.93 | 1.00 | 0.93 |
| **BBF3** | 878 | 4.49 | 0.95 | 0.66 |  | 1590 | 6.79 | 1.00 | 0.92 |
| **ABBF1** | 844 | 4.57 | 0.96 | 0.68 |  | 3731 | 7.50 | 1.00 | 0.91 |
| **ABBF2** | 991 | 5.00 | 0.98 | 0.72 |  | 1942 | 7.01 | 1.00 | 0.93 |
| **ABBF3** | 985 | 4.91 | 0.98 | 0.71 |  | 1554 | 6.82 | 1.00 | 0.93 |

# Supplementary Figures

| A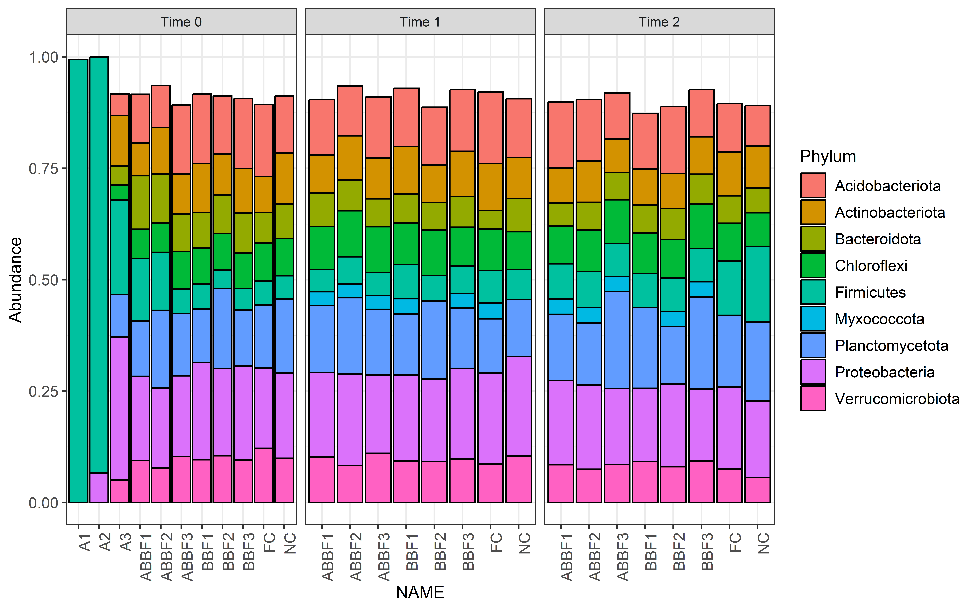 | B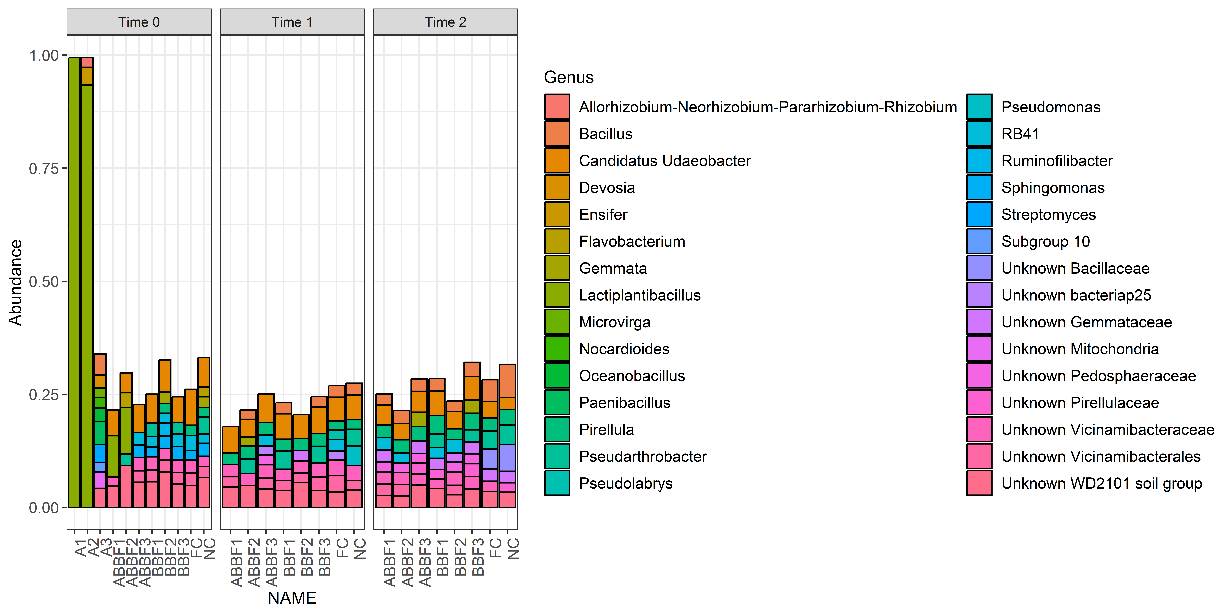 |
| --- | --- |
| C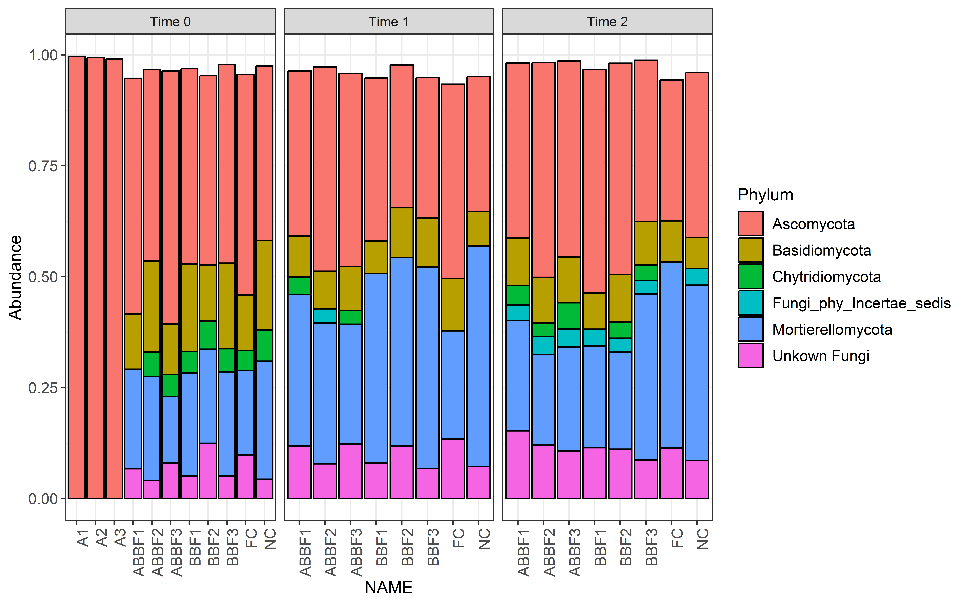 | D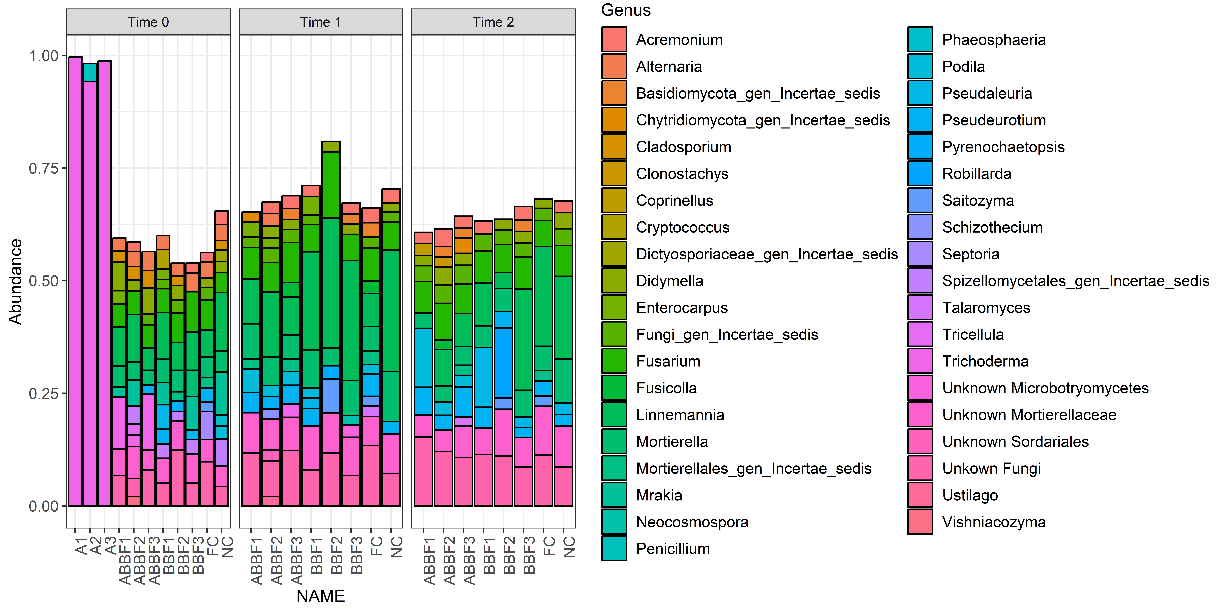 |
| E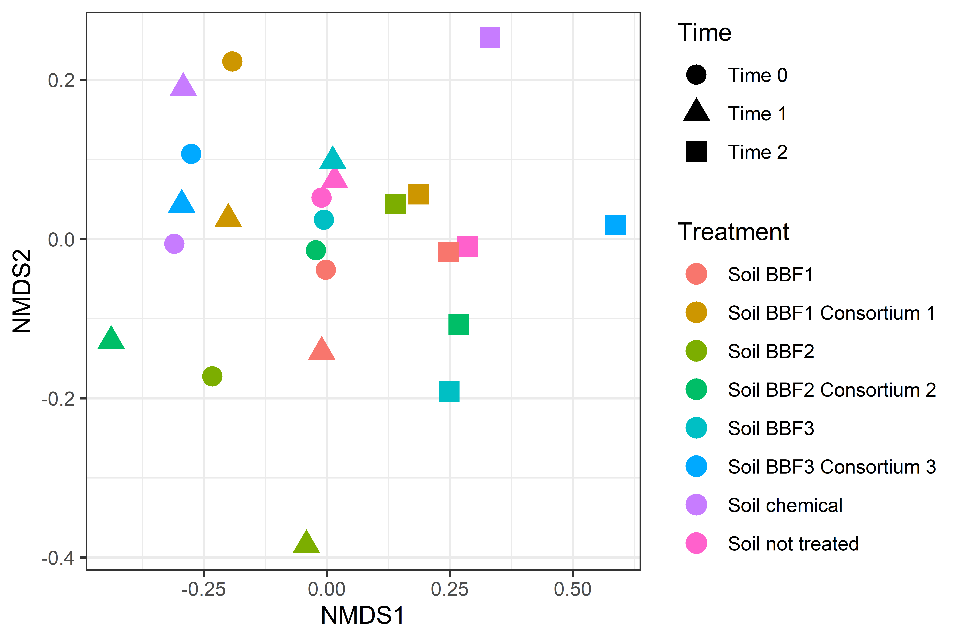 | F  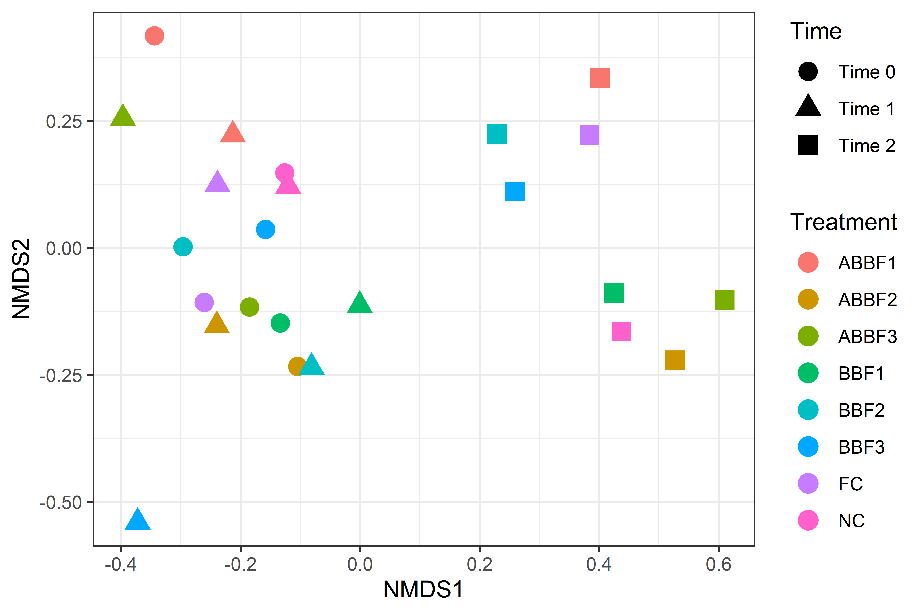 |

**Fig. S1.** Bar-plots of bacterial communities’ composition at phylum (A) and genus (B). Bar-plots of eukaryotic communities’ composition at phylum (C) and genus (D). Relative abundances are shown for each bar with a cut-off > 3% for phyla and >2% for genera. Non-metric multidimensional scaling (NMDS) ordination plots based on Bray-Curtis distances betw
